# Global, regional, and national burden of high body-mass index-related cancers and associated preventable life expectancy loss from 1990 to 2021

**DOI:** 10.3389/fnut.2025.1641276

**Published:** 2025-08-19

**Authors:** Ling Huang, Dongdong Wang, Pinyi Li, Xinger Liang

**Affiliations:** ^1^Department of Gynaecology, Guangdong Women and Children Hospital, Guangzhou, China; ^2^Department of Pathology, Guangdong Women and Children Hospital, Guangzhou, China; ^3^College of Basic Medicine, Jinan University, Guangzhou, China

**Keywords:** cancer, high BMI, death, disability-adjusted life years, life expectancy

## Abstract

**Background:**

A high Body Mass Index (BMI) is a key modifiable risk factor for cancer incidence. Therefore, it is essential to monitor the evolving trends and impact of cancer linked to high BMI and to formulate suitable strategies to address this issue. This study aims to analyze, from 1990 to 2021, the burden and temporal trends of cancer attributable to BMI as well as their impact on life expectancy, with major patterns highlighted by sex, socio-demographic index (SDI), and geographical region.

**Methods:**

Utilizing data from the Global Burden of Disease Study 2021, which incorporates 328,938 data sources, we conducted a comprehensive analysis of cancer attributable to high BMI, specifically investigating mortality rates and disability-adjusted life years (DALYs) from 1990 to 2021. Age-standardized rates were used to facilitate cross-regional comparisons, accounting for differences in population size and demographics. The Socio-demographic Index (SDI) was employed to categorize regions and evaluate correlations between cancer burden and economic development. In addition, we used the abridged period life table to estimate the impact of high BMI-related cancer on life expectancy.

**Results:**

The age-standardized death and DALY rates of cancer linked to high BMI increased (average annual percent change, 0.4 (95% UI: 0.4–0.5) for mortality and 0.5 (95% UI: 0.4–0.6) for DALY) from 1990 to 2021. The age-standardized death and DALY rates of cancer associated with high BMI were higher in females than in males globally. The alarming proportional increase in deaths from the main cancer associated with high BMI was observed among younger age groups (<45 years) from 1990 to 2021. In 2021, the most significant increases in life expectancy at birth were observed in regions with a high socio-demographic index, with gains of 0.12 (95% CI: −0.45–0.69) years for males and 0.19 (95% CI: −0.35–0.73) years for females, respectively. It is predicted that the age-standardized death rate of cancer attributable to high BMI will increase from 3.31 (3.29–3.33) in 2021 to 3.32 (1.27–5.37) by 2046 in males, and from 4.36 (4.38–4.39) to 4.90 (1.96–7.86) in females.

**Conclusion:**

The age-standardized mortality and DALY rates of cancer linked to high BMI have increased substantially, with significant differences across sex, geographic region, and SDI. Interventions aimed at reducing exposure are crucial not only to mitigate the cancer burden attributable to high BMI effectively, but also to yield modest gains in life expectancy.

## Introduction

A high body mass index (BMI) is a significant global health concern. Over the past few decades, there has been a dramatic increase in the number of people with obesity, driven by the widespread adoption of Westernized diets and sedentary lifestyles (1). A study by Katrina F Brown et al. used data from nationally representative population surveys of the United Kingdom in 2015. The results showed that tobacco smoking and being overweight or obese remained the leading risk factors for risk-factor-attributable cancer cases (2). In a large-scale population-based study of 2.3 million Israeli adolescents, Furer et al. (3) found a positive correlation between adolescent high BMI and elevated cancer-related mortality in both sexes and across all cancer types over the following decade. The convergence of high BMI as a modifiable cancer risk factor and the nearly concurrent rise in both high BMI and cancer incidence strongly suggest that high BMI plays a pivotal role in the initiation and progression of cancer (4). High-BMI-related cancer has been reported to be associated with an increased risk of death (5). Professional indicators such as relative risk and absolute lifetime risk may be difficult for the general public to understand. Life expectancy (LE) is a synthetic measure that reflects the prevailing force of mortality across all subsequent ages. It is an absolute quantitative measure that is more intuitive and commonly used for setting public health priorities (6, 7). It is imperative to monitor the evolving trends and the impact of cancer attributable to high BMI on LE at the global, regional, and national scales. Scrutinizing trends in mortality rates and the epidemiological profiles of different countries will facilitate the development of appropriate strategies to address this situation.

This study aimed to examine the global, regional, and national effect of high BMI on LE of patients with cancer across 204 countries and regions from 1990 to 2021. This study seeks to assess the trends in cancer mortality and DALY rates over the past three decades at these levels, thereby facilitating progress tracking, resource allocation planning. Although Zhi et al. and Darren Jun Hao Tan et al. analyzed the global burden and temporal trends of cancer attributable to high BMI using data from the Global Burden of Disease Study 2019 (8, 9), an updated analysis is necessary 2 years later, especially considering the profound impact of the coronavirus disease 2019 pandemic on global public health resources. Notably, few studies have assessed the impact of cancer on gains in LE (10), our study has provided a comprehensive quantification of the effect of high BMI–associated modifiable risk factors on LE.

## Materials and methods

### Data collection

This is an ecological, population-level, secondary-data analysis of the Global Burden of Disease Study 2021, conducted in accordance with the Guidelines for Accurate and Transparent Health Estimates Reporting (GATHER). Released in May 2024, the GBD 2021 covers 204 countries and territories, providing data from 1990 to 2021. The study, which leverages 328,938 data sources, reveals health disparities across age, sex, location, and socioeconomic groups. Most projects utilize data from population-based cancer registries, the vital registration systems, and verbal autopsy studies, frequently sourced through the expansive GBD Collaborator Network. The GBD 2021 study estimated relevant metrics for 23 age groups from birth to ≥95 years, for males, females, and all sexes combined, and for 204 countries and territories grouped into 21 regions and seven super-regions. In contrast to GBD 2019, GBD 2021 refined the mediation rules by adding 87 and removing 64 pairs to reach a total of 158, and introduced the BPRF/star-rating system across 211 risk–outcome pairs to assess effect magnitude and evidence consistency, enabling clearer and more conservative burden estimates while flagging high-confidence (≥3-star) relationships. Vital registration and verbal autopsy data completeness—a source-specific estimate of the percentage of total cause-specific deaths reported in a given location and year—were assessed by location-year, and sources with less than 50% completeness were excluded. The GBD 2021 excluded 142 country-years of data because of their completeness. As with garbage codes, the GBD 2021 applied a 5% buffer so that sources included in the previous GBD cycle would not be excluded from the current cycle if they had at least 45% completeness, allowing the study to retain 24 country-years that had previously been dropped. The study then multiplied the estimated all-cause mortality for each age-sex-location-year by the cause-specific fraction for the corresponding age-sex-location-year to adjust all included sources to 100% completeness. Data collection and processing methods were reported in the previous publications. Advanced statistical models, such as meta-regression Bayesian, regularized, trimmed (MR-BRT) and DisMod-MR 2.1, are employed for analysis. Disease terms use standardized International Classification of Diseases (ICD) code (11–13). In GBD 2021, cancer included both malignant and benign tumors classified according to the International Classification of Diseases. The GBD comparative risk assessment methodology quantifies the proportion of each cancer type attributable to high BMI by analyzing exposure distributions, relative risks, and counterfactual risk scenarios (14).

All data utilized in this study are openly accessible through the online platform of the Global Health Data Exchange, available at https://ghdx.healthdata.org/. The search criteria specified the GBD risk category ‘risk factor’, focusing on high BMI as the exposure and on mortality and disability-adjusted life-years (DALYs) as outcomes. Within the four-level GBD hierarchy, we selected Level-2 Neoplasms under the Level-1 category non-communicable diseases. Neoplasms comprises 12 cancer types: colon and rectum cancer, liver cancer, thyroid cancer, gallbladder and biliary tract cancer, leukemia, kidney cancer, non-Hodgkin lymphoma, multiple myeloma, pancreatic cancer, breast cancer, ovarian cancer, and uterine cancer. The scope encompassed all geographical regions from 1990 to 2021. The metrics included numbers and rates, and the sex categories included male, female, and both.

### Statistical analysis overview

Using data from the Global Burden of Diseases (GBD) database, we conducted a comprehensive analysis of cancer cases attributable to high BMI, specifically examining mortality and DALY rates at global, regional, and national scales from 1990 to 2021. Key metrics, including the age-standardized death rate (ASDR) and age-standardized DALY rate, were computed for each level, and the corresponding world maps were generated to visualize these rates. All choropleth and dot-density world maps were produced in R software using the sf and ggplot2 packages. All visualizations were exported at 300 dpi. DALYs are defined as the sum of years of life lost due to premature mortality and years lived with disability. The age-standardized death rate (ASDR) is the weighted average of age-specific death rates in a study population, calculated using the specified standard population age distribution as weights; this removes the confounding effect of differing age structures across populations or over time, thereby enabling unbiased comparisons of underlying mortality risk (11).

High body-mass index (BMI) for adults (ages 20+) is defined as BMI of 20–23 kg/m^2^. High BMI for children and adolescents (ages 2–19) is defined as being overweight or obese based on International Obesity Task Force standards. Population-based studies reporting mean BMI or overweight/obesity prevalence after January 1, 1980, were included in the study. For adults, studies were included if they defined overweight as BMI ≥ 25 kg/m^2^ and obesity as BMI ≥ 30 kg/m^2^. For children (ages 2–19), studies were included if they used IOTF standards to define overweight and obesity thresholds. Non-random studies, special subpopulations, alternative adiposity measures, *n* < 20 per age-sex group, reviews, and non-English-language articles were excluded (11).

To assess trends over time, the estimated annual percentage change (EAPC) was derived from a regression model that fits the natural logarithm of the rate to the calendar year. This is described by the equation Y = *α* + *β*X + *ϵ*, where Y = ln(rate), X = calendar year, and ϵ = error term. The EAPC was then calculated using the formula EAPC = 100 × (exp(β) − 1). The confidence intervals (CI) for EAPC were obtained from the linear model. World maps were created to depict the EAPC for these metrics. We determined the average annual percentage change (AAPC) and corresponding 95% CI for the period from 1990 to 2019. This was performed to ascertain whether the temporal trends significantly deviated from zero at a significance level of 0.05 (12, 15). Joinpoint regression was performed with Joinpoint 5.0 under a log-linear Poisson model, using ln(rate) as the outcome. The number of joinpoints was determined by a permutation test (Monte Carlo, 4,499 replicates, with an overall *α* of 0.05), starting from zero up to a maximum of five joinpoints. Model selection followed the Bayesian Information Criterion when several configurations were statistically equivalent. The resulting piecewise model was integrated to compute the AAPC as the geometric weighted mean of segment-specific annual percent changes across the entire study period. The EAPC is the constant annual rate of change estimated from a single log-linear regression over the entire study period, assuming a uniform exponential trend. The AAPC synthesizes the period by computing a weighted geometric mean of segment-specific annual percentage changes from the piecewise model, thereby summarizing non-linear trends while retaining an annualized metric (11).

We utilized the abbreviated period life table to compute life expectancy (LE) stratified by age brackets and gender for the years 1990 and 2021 (16). The analysis encompassed age groups from 0 to 1 year, 1–4 years, and subsequent 16 five-year bands for individuals aged 5 to 84 years, with the final category including those 85 years and older. The LE for each demographic segment was determined using the formula: LE = ΣLX/lx, where ΣLX represents the cumulative person-years of survival for individuals aged X and above, and lx denotes the number of survivors in age group X. Cause-eliminated life expectancy (CELE) was defined as the life expectancy if cancer attributable to high BMI deaths were eliminated and was calculated based on cancer attributable to high BMI-eliminated mortality rate (17).

For a more granular analysis, patients with cancer attributable to high BMI were stratified into age groups with five-year intervals. An analysis was then performed to examine the age distribution of the different types of cancer attributable to high BMI mortality in 1990 and 2021 at a global level.

The Bayesian Age-Period-Cohort (BAPC) model, recognized for its enhanced predictive accuracy, has been successfully implemented in several scholarly works (13, 14). The model was fitted with the R package “INLA” using integrated nested Laplace approximation. Age, period, and cohort effects were assigned second-order random-walk priors on the log scale; the precision hyper-priors followed Gamma (1, 0.00005) to ensure weak informativeness. Model convergence was assessed via trace plots, the Gelman–Rubin potential scale reduction factor (less than 1.01), and the effective sample size (greater than 400). Posterior predictive checks (Bayesian *p*-values 0.36 to 0.63) and WAIC comparisons against a first-order random-walk alternative confirmed an adequate fit (18, 19). Utilizing the BAPC package within the RStudio environment, we projected the ASDR up to the year 2046.

All statistical analyses were conducted using Joinpoint version 5.0 and R version 4.4.2. All statistical tests were two-sided, with a significance level set at *p* < 0.05.

### Ethics statement

This study was approved by the Academic Ethics Committee of Guangdong Women and Children Hospital. Because GBD 2021 uses de-identified, publicly available data, the informed consent and ethical review were waived for this study.

## Results

### Time and sex trends of cancer attributable to high BMI from 1990 to 2021

Globally, the ASDR (per 100,000) attributable to a high BMI was 3.7 (95% uncertainty interval [UI]: 1.5–6.0) in 1990. The age-standardized DALY rate (per 100,000) of cancer attributable to high BMI was 87.5 (95% UI: 37.4–141.8) worldwide in 1990. From 1990 to 2021, the ASDR and DALY rate attributable to high BMI increased, with an AAPC of 0.4 (95% UI: 0.4–0.5) for death and 0.5 (95% UI: 0.4–0.6) for DALY. Notably, the ASDR and DALY rate of cancer attributed to high BMI were higher in women than in men globally. However, growth rates were higher in men than in women during this period (Table 1).

**Table 1:** The age-standardized death and DALY rate of cancer attributable to high BMI in 1990 and 2021 and their variations from 1990 to 2021.

	1990		2021		1990–2021	
	Age-standardized death rate (per 100,000)	Age-standardized DALY rate (per 100,000)	Age-standardized death rate (per 100,000)	Age-standardized DALY rate (per 100,000)	AAPC of death rate (95% UI, %)	AAPC of age-standardized DALY rate (95% UI, %)
Global	3.7 (1.5–6)	87.5 (37.4–141.8)	4.2 (1.7–6.8)	102.2 (43.2–165)	0.4 (0.4–0.5)	0.5 (0.4–0.6)
Sex						
Female	4.4 (1.7–7.2)	105.3 (43.4–171.6)	4.7 (1.8–7.7)	114.6 (46.1–185.9)	0.2 (0.1–0.3)	0.3 (0.2–0.4)
Male	2.7 (1.3–4.4)	66.7 (31.4–106.6)	3.6 (1.6–5.8)	88.2 (39.4–143.4)	0.9 (0.8–1.0)	0.9 (0.8–1.0)
SDI						
High SDI	6.1 (2.4–10.1)	146.1 (58.5–239.4)	6 (2.4–9.9)	146.2 (59.7–237.6)	−0.0 (−0.2–0.1)	−0.0 (−0.17–0.13)
High-middle SDI	4.5 (1.9–7.4)	114.1 (50.1–185)	5.3 (2.2–8.7)	132.3 (55.3–215.1)	0.5 (0.4–0.7)	0.5 (0.3–0.7)
Middle SDI	1.7 (0.8–2.7)	44.7 (22.4–70.8)	3 (1.3–4.9)	80.5 (35.3–129.8)	1.9 (1.8–2.0)	1.9 (1.8–2.0)
Low-middle SDI	1.1 (0.5–1.6)	28.1 (14.2–42.8)	2.2 (1–3.6)	59.9 (26.4–95.4)	2.5 (2.4–2.6)	2.5 (2.4–2.6)
Low SDI	0.9 (0.4–1.5)	25.5 (12–39.5)	1.6 (0.7–2.6)	42.2 (18.4–67.3)	1.7 (1.7–1.8)	1.6 (1.6–1.7)
GBD super regions						
Southeast Asia, East Asia, and Oceania	1.3 (0.6–2)	37.3 (19.5–57.8)	2.7 (1.2–4.5)	76 (32.4–125)	2.5 (2.3–2.7)	2.3 (2.2–2.5)
Central Europe, Eastern Europe, and Central Asia	6.4 (2.7–10.2)	164.3 (71.9–260.7)	8.2 (3.4–13.4)	200.6 (84–328.3)	0.8 (0.6–1.1)	0.7 (0.4–0.9)
Latin America and Caribbean	3.4 (1.5–5.5)	146.3 (57.9–241.3)	5.2 (2.2–8.6)	146.3 (59.1–238.4)	1.4 (1.2–1.5)	0 (−0.1–0.1)
High-income	6.1 (2.4–10.1)	12.8 (7–19.3)	6.1 (2.4–10)	32.9 (15.3–51.1)	0 (−0.1–0.1)	3.1 (3–3.3)
Sub-Saharan Africa	1.5 (0.6–2.5)	87.4 (37.4–141.7)	3 (1–5)	102.1 (43.2–164.9)	2.2 (2–2.3)	0.5 (0.4–0.6)
North Africa and Middle East	3.1 (1.5–4.9)	82.9 (39.9–129.2)	5.3 (2.3–8.7)	135.8 (60.1–216.9)	1.7 (1.7–1.8)	1.6 (1.5–1.7)
South Asia	0.5 (0.3–0.7)	88.2 (41.7–141.4)	1.2 (0.5–1.9)	134.3 (59.6–219.8)	3.1 (2.9–3.3)	1.4 (1.2–1.6)
World Bank Regions	2.5 (1–4)	39.4 (15.6–62.6)	4.2 (1.7–6.8)	74.6 (26.3–123.4)	0.2 (−0.5–1)	2.1 (2–2.2)
European Union	6.7 (2.7–11)	87.6 (37.4–141.9)	6.7 (2.6–11.4)	102 (43.2–164.6)	0 (−0.1–0.1)	0.5 (0.4–0.6)
WHO region	3.7 (1.5–6)	156.8 (64.6–257.7)	4.2 (1.7–6.8)	155.1 (61.6–258.8)	0.4 (0.3–0.5)	−0.1 (−0.1–0)
League of Arab states	2.8 (1.3–4.5)	74.5 (35.7–115.8)	5.4 (2.3–8.5)	136.7 (60.5–215.9)	2.1 (1.9–2.3)	2 (1.8–2.2)
Commonwealth	2.1 (0.8–3.5)	46.1 (18.3–74.9)	2.6 (1–4.2)	59.9 (24.4–95.7)	0.7 (0.5–0.8)	0.8 (0.7–1)
OECD Countries	6.1 (2.4–10.2)	148 (59.8–241.4)	6.2 (2.5–10.2)	151.2 (62.2–244.7)	0 (0–0.1)	0.1 (0–0.1)
G20	3.9 (1.6–6.5)	93 (39.4–152.2)	4.3 (1.7–7)	103.9 (43.7–168.4)	0.3 (0.2–0.4)	0.4 (0.2–0.5)
Nordic Region	5.7 (2.2–9.6)	47.2 (19.7–73.6)	5.3 (2–9.1)	92.7 (36.7–151.3)	−0.2 (−0.3--0.1)	2.2 (2.1–2.3)
World Bank Income Levels	3.7 (1.5–6)	87.4 (37.4–141.7)	4.2 (1.7–6.8)	102.1 (43.2–164.9)	0.4 (0.4–0.5)	0.5 (0.4–0.6)
Four World Regions	3.7 (1.5–6)	134.8 (51.9–226)	4.2 (1.7–6.8)	118.2 (45.2–198)	0.4 (0.4–0.5)	−0.4 (−0.5--0.3)
Association of Southeast Asian Nations	1 (0.5–1.6)	87.5 (37.4–141.7)	2.3 (1–3.9)	102.1 (43.2–164.9)	2.7 (2.6–2.7)	0.5 (0.4–0.6)
African Union	1.8 (0.7–2.9)	31.2 (15.6–48.7)	3.7 (1.4–6.1)	67.2 (28.5–110.4)	2.3 (2.2–2.4)	2.5 (2.4–2.6)
Organization of Islamic Cooperation	1.9 (0.9–3.1)	82.2 (36.8–133.4)	3.4 (1.4–5.5)	155.3 (68.2–250.4)	1.8 (1.7–2)	2.1 (1.8–2.4)
Gulf Cooperation Council	3.1 (1.4–5.1)	52.3 (24.8–82)	6.2 (2.7–9.9)	88.9 (37.7–142.8)	2.3 (2–2.5)	1.7 (1.6–1.8)
Sahel Region	1.4 (0.5–2.2)	36.5 (14–57.9)	2.3 (0.8–3.8)	60 (21.6–97.4)	1.7 (1.6–1.8)	1.6 (1.6–1.6)
Health System Grouping Levels	3.7 (1.5–6)	87.5 (37.4–141.7)	4.2 (1.7–6.8)	102.1 (43.2–164.9)	0.4 (0.4–0.5)	0.5 (0.4–0.6)

AAPC, average annual percentage change; DALY, disability-adjusted life-years; UI, uncertainty interval; SDI, socio-demographic index; GBD, global disease burden; WHO, World Health Organization; OECD, Organization for Economic Co-operation and Development; G20, Group of 20.

The rates of cancer death and DALYs attributable to high BMI across different SDI levels are summarized in Table 1. The results demonstrate that high-SDI regions still had the highest rates of cancer-related deaths and DALYs attributable to high BMI in 2021, with rates of 6.0 (95% UI: 2.4–9.9) for deaths and 146.2 (95% UI: 59.7–237.6) for DALYs. However, the growth rate in the high-SDI regions was the lowest during 1990–2021. In contrast, the low-middle-SDI region experienced the fastest growth in terms of ASDR and DALY rate during this period, with an AAPC of 2.5% for both death and DALY.

In 2021, Central Europe, Eastern Europe, and Central Asia recorded the highest rates of age-standardized cancer-related deaths and DALYs attributable to high BMI among the GBD super regions. However, from 1990 to 2021, the greatest percentage increase in cancer deaths attributable to high BMI 3.1 (95% UI: 2.9–3.3) was observed in South Asia, while the greatest percentage increase in DALYs 3.1 (95% UI: 3–3.3) was in high-income regions.

During the same period, the ASDR of cancers attributable to high BMI increased in all super GBD regions, except in high-income regions and European Union countries, where the rates remained stable. Similarly, age-standardized DALY rates increased in all super GBD regions from 1990 to 2021, except for Latin America and the Caribbean, the World Health Organization (WHO) region, and the four World Regions, where they decreased (Table 1).

### Age attributions of the cancer mortality burden related to high BMI from 1990 to 2021

**Figure 1:**
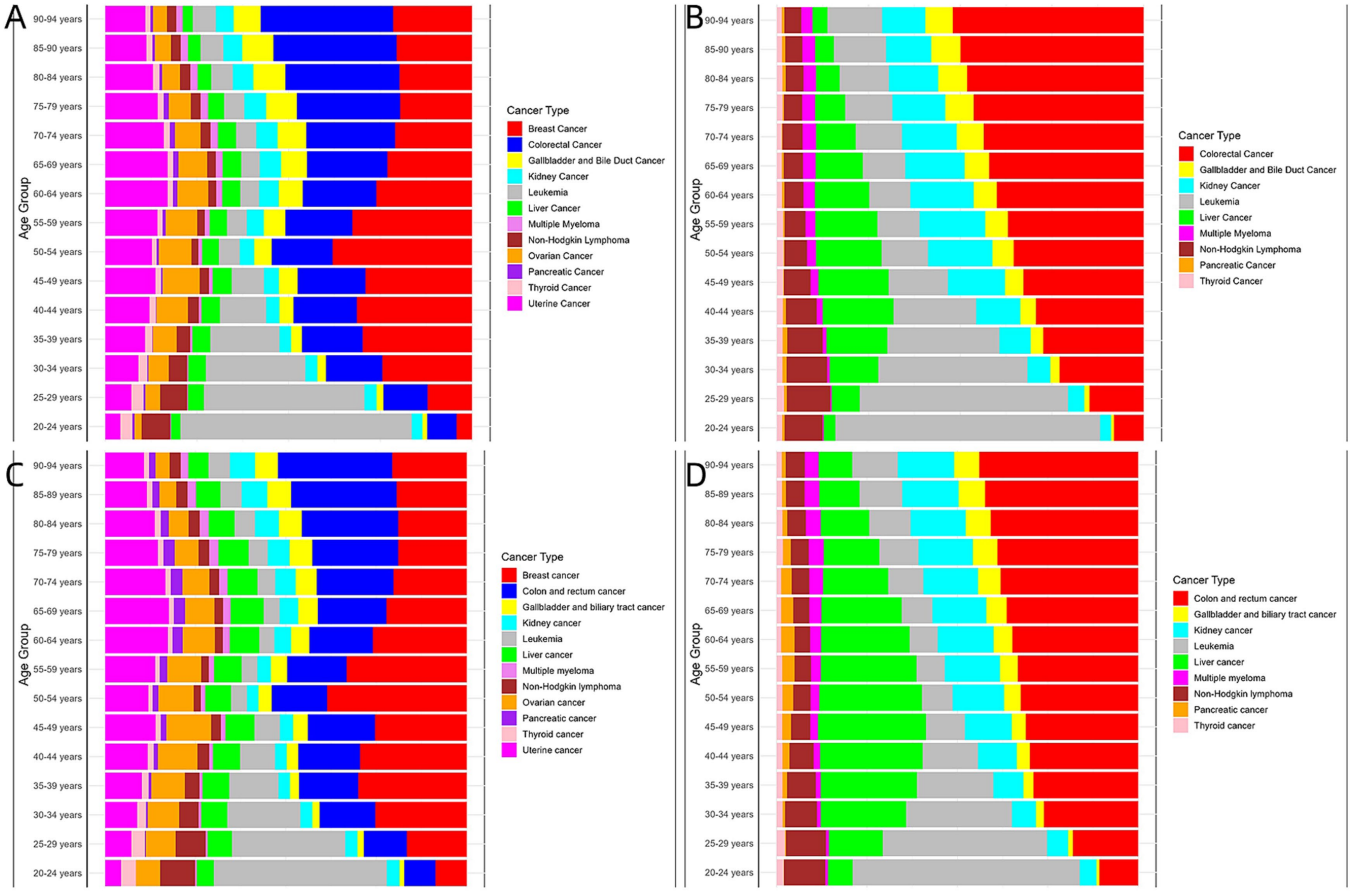
Age distribution and relative mortality proportion by cancer type for all deaths attributable to high BMI in 1990 and 2021. **(A)** Female, 1990. **(B)** Male, 1990. **(C)** Female, 2021. **(D)** Male, 2021.

To elucidate these trends across age groups, we analyzed mortality data starting at age 20, stratifying every 5 years into distinct age groups, resulting in a total of 16 age groups. Figure 1 illustrates the global distribution of cancer deaths attributable to high BMI by sex and age group and highlights the significant impact on younger populations of the main cancer related to high BMI over the past three decades.

Among women aged 35–69 years, breast cancer emerged as the leading cause of cancer-related deaths associated with a high BMI in 1990. In 2021, breast cancer mortality notably shifted toward younger age groups, with a significant increase in premature deaths under the age of 40 years compared with 1990 (29.87% (95% CI: 29.34–30.40%) in 2021 compared to 9.80% (95% CI: 9.45 –10.15%) in 1990). A striking finding was the doubling of breast cancer-related deaths among women aged 20–24 years from 1990 to 2021 (9.0% (95% CI: 5.1–12.8%) in 2021 compared to 4.3% (95% CI: 1.6–7.4%) in 1990). Similarly, among male patients aged > 40 years, colon and rectal cancers emerged as the leading causes of cancer-related deaths associated with a high BMI in 1990. Among them, the proportion of premature deaths under the age of 45 years caused by colon and rectal cancer was significantly higher in 2021 than in 1990 (31.36% (95% CI: 30.81 –31.91%) in 2021 compared to 20.84% (95% CI: 20.23 −21.45%) in 1990) (Figure 1).

### Regional patterns of the cancer mortality burden attributable to high BMI from 1990 to 2021

**Figure 2:**
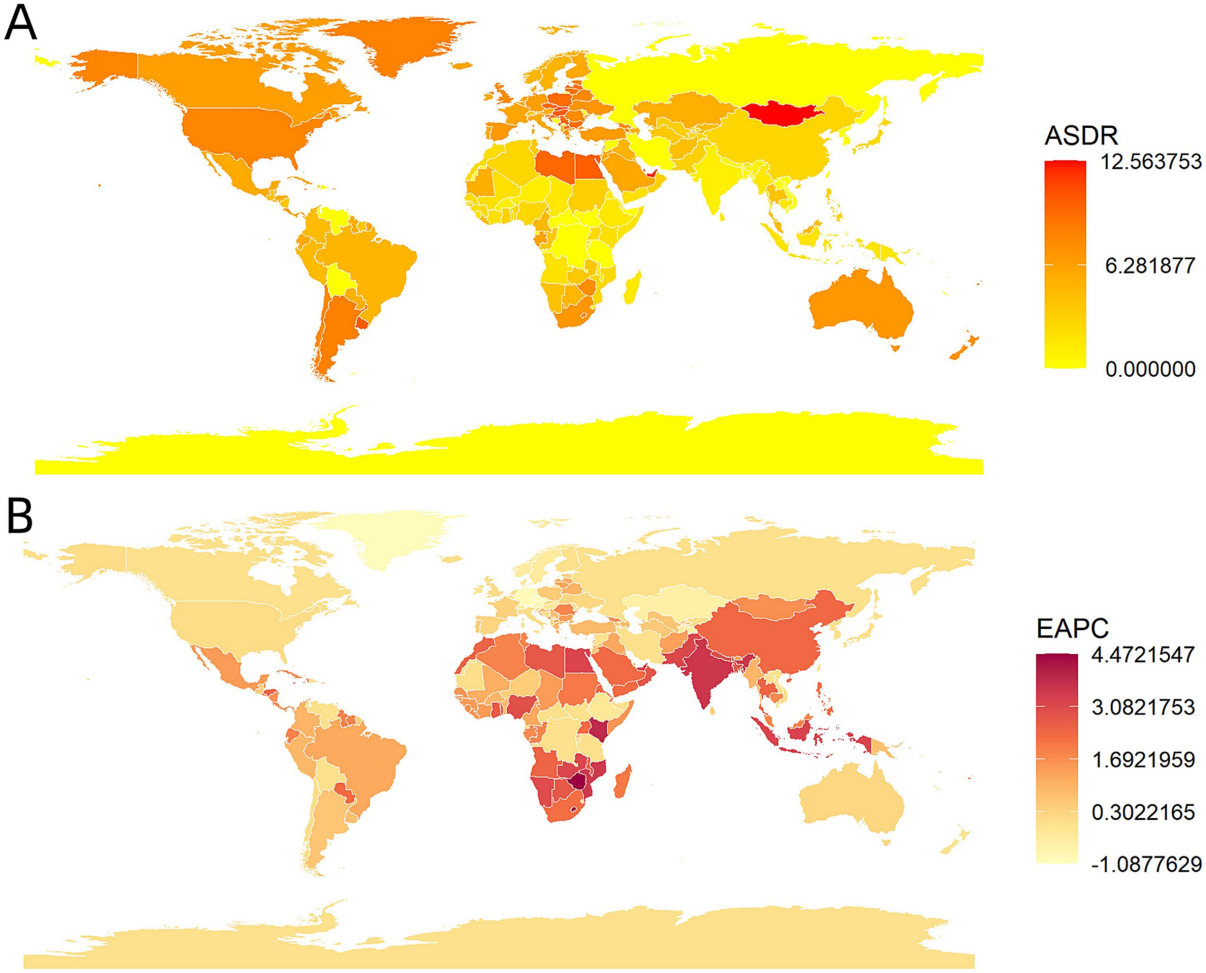
Age-standardized death rate (ASDR) and estimated annual percentage change (EAPC) of ASDR of the cancer attributable to high BMI burden by countries and territories. **(A)** ASDR, 2021. **(B)** EAPC, 1990 to 2021.

Figure 2 presents the age-standardized death rate (ASDR) and estimated annual percentage change (EAPC) across 204 countries and territories. From 1990 to 2021, Zimbabwe, Lesotho, and Kenya experienced the most pronounced increases in cancer mortality attributable to high BMI. Among these 204 countries and territories, Mongolia, the Kingdom of Tonga, and the Kingdom of Eswatini recorded the highest ASDR in 2021, with rates of 12.6 (95% UI: 5.1–21.0), 12.2 (95% UI: 3.9–22.6), and 12.1 (95% UI: 4.6–20.9) per 100,000 population, respectively. The most substantial increases in the ASDR from 1990 to 2021 occurred in Zimbabwe, Lesotho, and Kenya, with EAPC values of 4.47 (95% UI: 3.80–5.15), 4.28 (95% UI: 3.80–4.76), and 3.80 (95% UI: 3.66–3.95), respectively (Figure 2). Conversely, the most pronounced decreases in ASDR were observed in Czechia, Greenland, and Germany, with EAPC values of-1.09 (95% UI: −1.24−−0.94), −1.00 (95% UI: −1.09−−0.91), and-0.92 (95% UI: −1.03−−0.81), respectively, over the same period.

In conclusion, the cancer mortality burden attributable to a high BMI exhibits significant national and regional heterogeneity. Specifically, 20 countries and territories demonstrated a downward trend in ASDR, while 169 countries and territories experienced a significant upward trend. In addition, the ASDR remained stable in 15 countries and territories (Figure 2).

### Regional patterns of the effect of cancer attributable to high BMI on LE in 1990 and 2021

**Figure 3:**
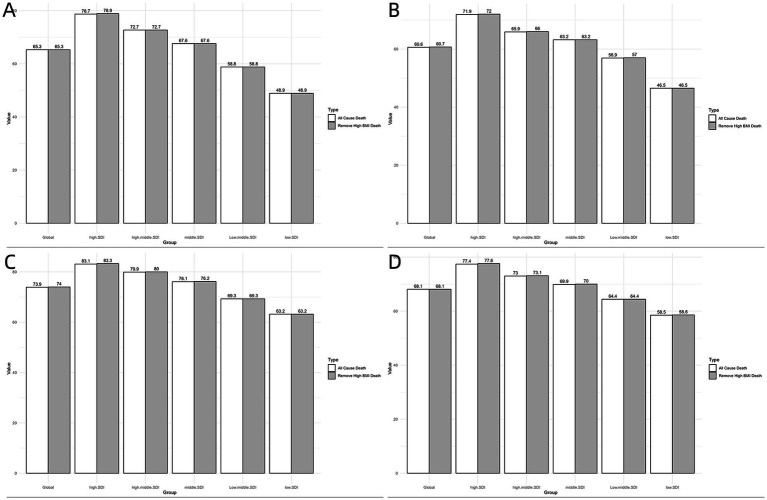
Life expectancy and cause eliminated life expectancy (CELE) of cancer death attributable to high BMI in different regions. **(A)** Females, 1990. **(B)** Males, 1990. **(C)** Females, 2021. **(D)** Males, 2021.

Figure 3 illustrates LE and CELE for cancer deaths attributable to high BMI across different regions in 1990 and 2021. Notably, the most substantial gains in LE occurred in high-SDI regions. In 2021, the estimated global LE at birth was 68.08 (95% CI: 67.84–68.32) years for men and 73.89 (95% CI: 73.64–74.13) years for women. If cancer deaths attributable to high BMI were eliminated, the LE would have increased to 68.15 (95% CI: 67.91–68.40) years for men and 73.98 (95% CI: 73.74–74.23) years for women, representing gains of 0.07 (95% CI: −0.27–0.41) years and 0.09 (95% CI: −0.26–0.43) years, respectively.

Regarding CELE in different SDI regions, the largest gains in LE at birth were seen in high-SDI regions for both sexes 0.12 (95% CI: −0.45–0.69) years for men and 0.19 (95% CI: −0.35–0.73) years for women in 2021. Conversely, the smallest gains were observed in low-SDI regions (0.01 (95% CI, −0.71–0.73) years for men and 0.03 (95% CI, −0.72–0.78) years for women). Significant heterogeneity was noted between the sexes, with women experiencing longer gains in LE than men upon the elimination of cancer attributable to high BMI.

In 1990, the estimated global LE at birth was 60.63 (95% CI: 60.34–60.91) years for men and 65.28 (95% CI: 64.98–65.58) years for women. If cancer deaths attributable to high BMI were eliminated, the LE would have increased to 60.66 (95% CI: 60.37–60.94) years for men and 65.34 (95% CI: 65.04–65.64) years for women, representing gains of 0.03 (95% CI: −0.76-0.82) years and 0.06 (95% CI: −0.77-0.89) years, respectively. Overall, the gains in LE are more pronounced in 2021 than in 1990 (Figure 3; Supplementary Tables 1–4).

### Time trends and projections of the global ASDR of cancer attributable to high BMI from 1990 to 2046

**Figure 4:**
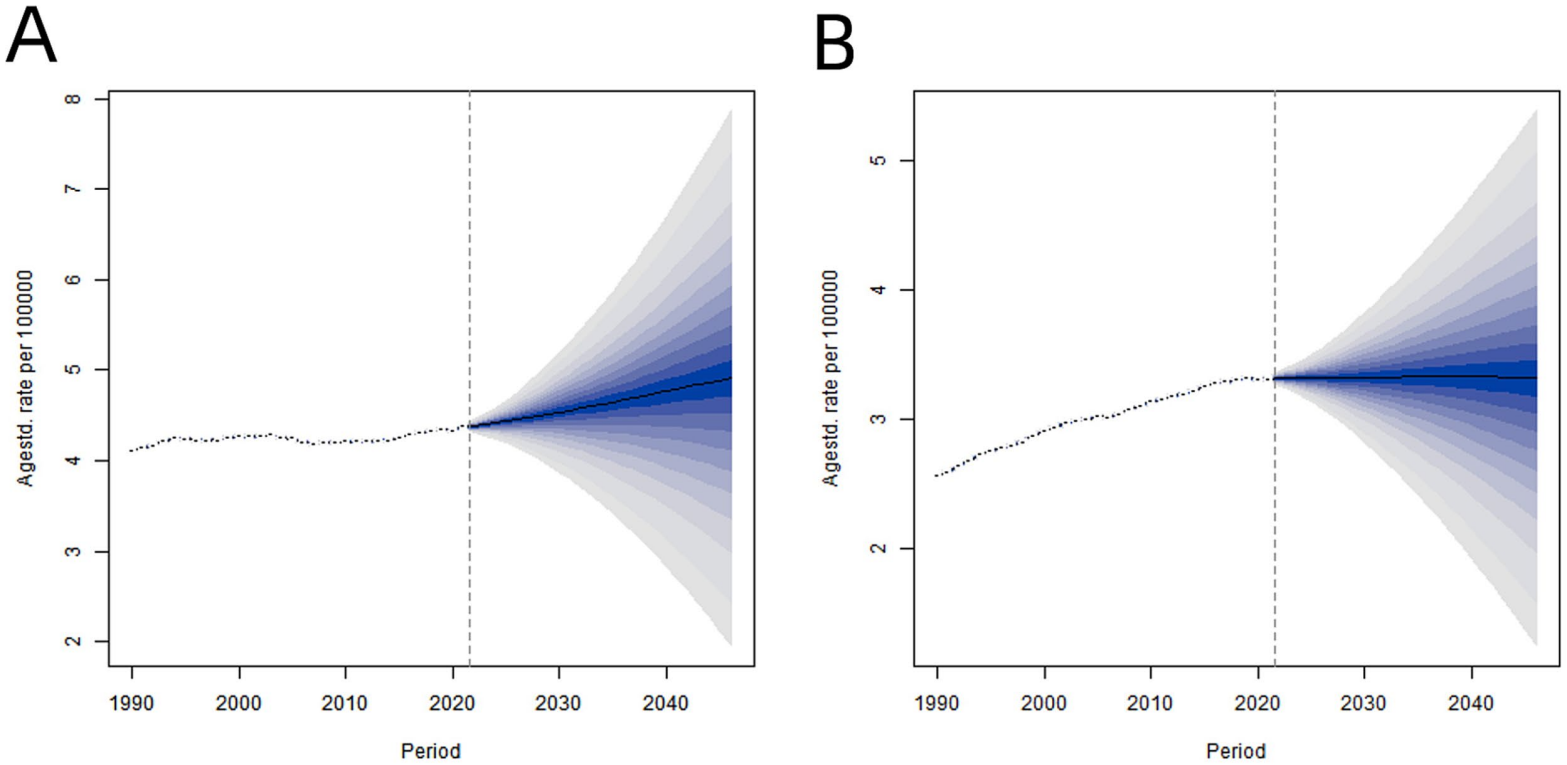
Time trend and projection of global age-standardized death rate of cancer attributable to high BMI from 1990 to 2046. **(A)** Females **(B)** Male.

Figure 4 illustrates the global age-standardized death rate (ASDR) trends and projections for cancer attributable to high BMI from 1990 to 2046, stratified by sex. The ASDR (per 100,000) of cancer attributable to high BMI will increase from 3.31 (3.29–3.33) in 2021 to 3.32 (1.27–5.37) by 2046 in males, and from 4.36 (4.38–4.39) to 4.90 (1.96–7.86) in females. It is predicted that, in the absence of effective interventions over the next 25 years, the ASDR of cancer attributable to high BMI is unlikely to decrease. Notably, the ASDR is anticipated to remain higher among women than men throughout this period (Figure 4).

## Discussion

With socioeconomic progress, cancer has emerged as a pervasive and borderless global health crisis. This malignant disease accounts for approximately 10 million lives annually (20). Obesity is widely recognized as a significant risk factor for various types of cancers (21). Consequently, the prevention and control of cancer attributable to high BMI, tailored to epidemic characteristics, precision, and local conditions, can significantly mitigate the burden of high BMI-related cancers. Unlike other studies that focused on analyzing morbidity and mortality rates, our research offers an in-depth examination of trends in high BMI-related cancer mortality across regions, countries, sexes, age groups, and potential gains in LE, all based on the GBD 2021 data.

Our findings revealed that the ASDR and DALY rate of cancer attributable to high BMI increased from 1990 to 2021. Over the past four decades, obesity has evolved from a localized epidemic to a global pandemic. This alarming trend is further highlighted by the consistent increase in the average BMI, a key metric for assessing obesity worldwide. For instance, the global average BMI, tracked from 1975 to 2014, climbed from 21.7 kg/m^2^ to 24.2 kg/m^2^ among men, and from 22.1 kg/m^2^ to 24.4 kg/m^2^ among women. During this period, the number of individuals classified as obese, based on a BMI of ≥30 kg/m^2^, more than tripled (22, 23). Various mechanisms by which high BMI affects cancer risk have been proposed and extensively studied (24–26). A study by Hong et al. (27) supported the notion that modifiable external exposure can influence tumor development by modulating tumor-related target genes and pathways. Most individuals with obesity have inflamed adipose tissue. Systemically, metabolic syndromes, including dyslipidemia and insulin resistance, often occur in the context of adipose tissue inflammation and collaborate with local mechanisms to sustain an inflamed microenvironment and promote tumor growth (28, 29). Moreover, the positive energy balance associated with obesity induces a variety of metabolic factors such as insulin and insulin-like growth factor-1, which alter the nutritional milieu and create an environment conducive to tumor initiation and progression (30). In addition to the mechanisms involved in carcinogenesis, high BMI promotes cancer progression, impairs the efficacy of cancer therapies, and contributes to the poor prognosis of patients with obesity with cancer through obesity-related comorbidities (31). Thus, the dramatic increase in obesity likely contributed to the increase in the ASDR and DALY rate of cancer attributable to high BMI.

Globally, the ASDR and DALY rate of cancer attributable to high BMI are higher in women than in men, consistent with the observed sex-related differences in obesity prevalence (23). The incidence of obesity is 14% in men and 18% in women (32). These marked disparities are evident across all global regions, highlighting distinct sexual dimorphism in obesity prevalence. This dimorphism is attributed to differences in underlying pathophysiology, including variations in fat distribution, energy metabolism, gut microbiota composition, and chromosomal and genetic influences (33).

The burden of cancer attributable to high BMI varies across different SDI regions. Since 1990, the regional ASDR and DALY rate of cancer attributable to high BMI have generally increased. The distribution of this disease burden parallels the prevalence of high BMI across regions. Reports have indicated that the prevalence of obesity is higher in high-income countries than in low-income countries (12, 34). Differences between the SDI regions may also be influenced by higher cancer detection rates in high-income countries that benefit from complete data coverage, cancer registry systems, and comprehensive datasets (35). We also observed a rapid increase in cancer burden in several low-SDI and low-middle-SDI regions, such as South Asia. This trend has been related to economic development in these regions over the past three decades. Inadequate healthcare and cancer prevention measures in these regions may contribute to the high mortality rate of cancer attributable to high BMI (36). To address these challenges, low-SDI regions could implement affordable interventions such as BMI screening, community nutrition programs, and primary care strengthening. Additionally, improving the data collection systems in low-SDI regions could enhance our understanding of the burden and risk factors, facilitating the design of more effective public health strategies with limited resources. High-and middle-SDI regions could enhance cancer screening programs to facilitate earlier detection and treatment of cancers attributable to high BMI. Furthermore, public health campaigns and community-based interventions should be carried out in high-and middle-SDI regions to mitigate the impact of high BMI on cancer incidence.

Countries with the highest ASDR include Mongolia. As a landlocked nation, Mongolia’s diet often contains excessive salt and fat and insufficient fiber (37), which may contribute to the increased incidence of cancers attributable to high BMI. Moreover, countries such as the Kingdom of Tonga exhibit exceptionally high obesity levels, largely because not enough fruits and vegetables are consumed while energy-dense, nutrient-poor options are readily available and cheap (38). Besides, healthcare provision in these countries is often inadequate, leading to many patients with cancer missing timely diagnosis or treatment. Therefore, nutritional education programs promoting a low-salt, low-fat, high-fiber diet should be implemented in these countries to reduce obesity and cancer risk from unhealthy diets.

Our study showed an alarming proportional increase in deaths from breast cancer attributable to high BMI among younger age groups in 2021. Most breast cancer screening guidelines, developed by international cancer associations or health management departments, recommend that screening should start at the age of 40 years or older (39). Few health management departments have focused on early screening and intervention for breast cancer in individuals aged < 40 years (40). Similarly, the proportional increase in deaths from colon and rectum cancer attributable to high BMI also affected younger male individuals. The trend toward earlier-onset cancers attributable to high BMI raises the hypothesis that young people should receive professional dietary and health guidance to maintain a healthy weight, and that medical institutions should be encouraged to offer early cancer diagnosis and treatment for this age group. However, given the ecological and observational nature of this study, this hypothesis needs confirmation through further large-scale prospective randomized controlled trials.

The gains in LE from the elimination of cancer attributable to high BMI were greater in women than in men. Regarding CELE in different SDI regions, the largest gains in LE were observed in regions with high SDI for both sexes in 2021. These substantial gains in the high-SDI regions are consistent with having the highest cancer mortality rates attributable to high BMI. Sex discrepancies in CELE may be driven by disparities in exposure distribution and genetic predisposition between sexes (41, 42).

Some studies have examined the burden of cancer attributable to high BMI. Zhi et al. (8) analyzed the global burden and temporal trends of cancer attributable to high BMI using data from the Global Burden of Disease Study 2019. Their research assessed the trends and magnitudes of the high BMI-related cancer burden by sex, SDI and geographical region. Darren Jun Hao Tan et al. (9) estimated global and regional temporal trends in the burden of cancer attributable to high BMI and the contributions of various cancer types. However, these studies did not incorporate the latest data, from 2019 to 2021. In contrast to previous research, disaggregating results into 5-year age bands reveals that the relative peak burden of several major cancers attributable to high BMI has shifted to younger age groups, thereby enabling targeted public health recommendations. Unlike previous studies that focused on cancer-specific DALYs, our study introduced the CELE to quantify the broader impact of high BMI-related cancers on population longevity.

The general incompleteness of the GBD database poses limitations to this study. The accuracy of the estimations may be constrained in countries with sparse cancer registry data, potentially affecting regional comparisons. Limited cancer registrations often exist in low-SDI regions, resulting in an underestimation of cancer mortality in these areas. Time limitations also affect the GBD 2021 database. Although the database is updated annually, the most recent data may not reflect recent disease trends and health risk factors owing to delays in some data sources. All estimates end in 2021 and therefore do not capture post-2021 dynamics, such as the COVID-19-related disruption of cancer services. Because our analysis is ecological in nature, observed associations between BMI and cancer at the population level may not hold for individuals. Consequently, recommendations for screening or clinical intervention should be interpreted with caution and validated through individual-level studies. Moreover, the GBD database has shortcomings in terms of regional specificity. For instance, cultural, social, and environmental factors in different regions may influence the disease burden, but the GBD database may not accurately capture these differences. When secondary data are used, potential sources of bias include under-reporting of cancer cases in low-income settings, misclassification of BMI in self-reported datasets, and regional heterogeneity in the quality of death certificates. Finally, despite being statistically significant, the modest gains in LE highlight the need to address multiple coexisting risk factors beyond BMI alone.

In this study, we demonstrated that the age-standardized mortality and DALY rates of cancers linked to high BMI increased substantially between 1990 and 2021, with significant variations by sex, geographic region, and SDI. The alarming proportional rise in high-BMI-attributable cancer deaths occurred predominantly among younger age groups over the same period. Interventions aimed at reducing exposure are therefore essential not only to curb the growing cancer burden linked to high BMI but also to yield modest gains in life expectancy.

## Data Availability

Publicly available datasets were analyzed in this study. This data can be found at: https://ghdx.healthdata.org/.

## References

[1] Fu Y Guo X Sun L Cui T Wang J Liu Y. Exploring the interplay of diet, obesity, immune function, and Cancer. Cancer Discov. (2024) 14:2047–50. doi: 10.1158/2159-8290.Cd-24-0834, PMID: 39485246

[2] Brown KF Rumgay H Dunlop C Ryan M Quartly F Cox A. The fraction of Cancer attributable to modifiable risk factors in England, Wales, Scotland, Northern Ireland, and the United Kingdom in 2015. Br J Cancer. (2018) 118:1130–41. doi: 10.1038/s41416-018-0029-6, PMID: 29567982 PMC5931106

[3] Furer A Afek A Sommer A Keinan-Boker L Derazne E Levi Z. Adolescent obesity and midlife Cancer risk: a population-based cohort study of 2·3 million adolescents in Israel. Lancet Diabetes Endocrinol. (2020) 8:216–25. doi: 10.1016/s2213-8587(20)30019-x, PMID: 32027851

[4] Zheng X Wang Y Chen Y Liu T Liu C Lin S. Metabolic obesity phenotypes and the risk of Cancer: a prospective study of the Kailuan cohort. Front Endocrinol. (2024) 15:1333488. doi: 10.3389/fendo.2024.1333488, PMID: 39479267 PMC11521940

[5] García-Witulski C. Non-communicable disease mortality and economic costs attributable to high body mass index in Argentina. Public Health. (2025) 238:139–51. doi: 10.1016/j.puhe.2024.10.034, PMID: 39662129

[6] Davies S. Life expectancy: Public health and politics. Lancet. (2025). doi: 10.1016/s0140-6736(25)01318-2 40578372

[7] Roubal AM Pollock EA Gennuso KP Blomme CK Givens ML. Comparative methodologic and practical considerations for life expectancy as a public health mortality measure. Public Health Rep. (2022) 137:255–62. doi: 10.1177/0033354921999407, PMID: 33706596 PMC8900236

[8] Zhi X Kuang XH Liu K Li J. The global burden and temporal trend of Cancer attributable to high body mass index: estimates from the global burden of disease study 2019. Front Nutr. (2022) 9:918330. doi: 10.3389/fnut.2022.918330, PMID: 35958256 PMC9360580

[9] Tan DJH Ng CH Muthiah M Yong JN Chee D Teng M. Rising global burden of Cancer attributable to high Bmi from 2010 to 2019. Metab Clin Exp. (2024) 152:155744. doi: 10.1016/j.metabol.2023.155744, PMID: 38029839 PMC11321712

[10] Syriopoulou E Bower H Andersson TM Lambert PC Rutherford MJ. Estimating the impact of a Cancer diagnosis on life expectancy by socio-economic Group for a Range of Cancer types in England. Br J Cancer. (2017) 117:1419–26. doi: 10.1038/bjc.2017.300, PMID: 28898233 PMC5672926

[11] GBD 2021 Risk Factors Collaborators. Global Burden and Strength of Evidence for 88 Risk Factors in 204 Countries and 811 Subnational Locations, 1990-2021: A Systematic Analysis for the Global Burden of Disease Study 2021. Lancet. (2024) 403:10440:2162–203. doi: 10.1016/s0140-6736(24)00933-4 PMC11120204 38762324

[12] Islam A Sultana H Nazmul Hassan Refat M Farhana Z Abdulbasah Kamil A Meshbahur RM. The global burden of overweight-obesity and its association with economic status, benefiting from steps survey of who member states: a Meta-analysis. Prev Med Rep. (2024) 46:102882. doi: 10.1016/j.pmedr.2024.102882, PMID: 39290257 PMC11406007

[13] Murray CJL. The global burden of disease study at 30 years. Nat Med. (2022) 28:2019–26. doi: 10.1038/s41591-022-01990-1, PMID: 36216939

[14] Zhu B Gu H Mao Z Beeraka NM Zhao X Anand MP. Global burden of Gynaecological cancers in 2022 and projections to 2050. J Glob Health. (2024) 14:04155. doi: 10.7189/jogh.14.04155, PMID: 39148469 PMC11327849

[15] Fay MP Tiwari RC Feuer EJ Zou Z. Estimating average annual percent change for disease rates without assuming constant change. Biometrics. (2006) 62:847–54. doi: 10.1111/j.1541-0420.2006.00528.x, PMID: 16984328

[16] Hsieh JJ. A general theory of life table construction and a precise abridged life table method. Biometr J Biometr Zeitschr. (1991) 33:143–62. doi: 10.1002/bimj.4710330204, PMID: 12343327

[17] Wang C Chang Y Ren J Wu Z Zheng Y Luo Z. Modifiable risk-attributable and age-related burden of lung Cancer in China, 1990-2019. Cancer. (2023) 129:2871–86. doi: 10.1002/cncr.34850, PMID: 37221876

[18] Knoll M Furkel J Debus J Abdollahi A Karch A Stock C. An R package for an integrated evaluation of statistical approaches to Cancer incidence projection. BMC Med Res Methodol. (2020) 20:257. doi: 10.1186/s12874-020-01133-5, PMID: 33059585 PMC7559591

[19] Wu B Li Y Shi B Zhang X Lai Y Cui F. Temporal trends of breast Cancer burden in the Western Pacific region from 1990 to 2044: implications from the global burden of disease study 2019. J Adv Res. (2024) 59:189–99. doi: 10.1016/j.jare.2023.07.003, PMID: 37422280 PMC11082062

[20] Bradley CJ Kitchen S Owsley KM. Working, low income, and Cancer caregiving: financial and mental health impacts. J Clin Oncol. (2023) 41:2939–48. doi: 10.1200/jco.22.02537, PMID: 37043714 PMC10414725

[21] Romero M Lian YF Piquer A Borràs-Ferré N Zorzano A Ivanova S. The impact of obesity and lipids on Cancer: insights into mechanisms and potential interventions. Semin Cancer Biol. (2025) 115:53–74. doi: 10.1016/j.semcancer.2025.07.006, PMID: 40716505

[22] NCD Risk Factor Collaboration (NCD-RisC). Trends in Adult Body-Mass Index in 200 Countries from 1975 to 2014: A Pooled Analysis of 1698 Population-Based Measurement Studies with 19·2 Million Participants. Lancet. (2016) 387:10026:1377–96. doi: 10.1016/s0140-6736(16)30054-x PMC7615134 27115820

[23] Afshin A Forouzanfar MH Reitsma MB Sur P Estep K Lee A. Health effects of overweight and obesity in 195 countries over 25 years. N Engl J Med. (2017) 377:13–27. doi: 10.1056/NEJMoa1614362, PMID: 28604169 PMC5477817

[24] Townsel A Jaffe M Wu Y Henry CJ Haynes KA. The epigenetic landscape of breast Cancer, metabolism, and obesity. Adv Exp Med Biol. (2024) 1465:37–53. doi: 10.1007/978-3-031-66686-5_3, PMID: 39586992

[25] Luo M Ma X Ye J. Reductive stress-a common metabolic feature of obesity and Cancer. Acta Pharm Sin B. (2024) 14:5181–5. doi: 10.1016/j.apsb.2024.08.034, PMID: 39807313 PMC11725146

[26] Li J Wang D Song S Wang Y Wu X Du Z. Analyzing the potential targets and mechanism of per-and Polyfluoroalkyl substances (Pfas) on breast Cancer by integrating network toxicology, single-cell sequencing, spatial Transcriptomics, and molecular simulation. Funct Integr Genomics. (2025) 25:119. doi: 10.1007/s10142-025-01616-y, PMID: 40465018

[27] Hong Y Wang D Liu Z Chen Y Wang Y Li J. Decoding per-and Polyfluoroalkyl substances (Pfas) in hepatocellular carcinoma: a multi-omics and computational toxicology approach. J Transl Med. (2025) 23:504. doi: 10.1186/s12967-025-06517-z, PMID: 40317014 PMC12049027

[28] Li J Xu N Hu L Xu J Huang Y Wang D. Chaperonin containing Tcp1 subunit 5 as a novel Pan-Cancer prognostic biomarker for tumor Stemness and immunotherapy response: insights from multi-omics data, integrated machine learning, and experimental validation. Cancer Immunol Immunother. (2025) 74:224. doi: 10.1007/s00262-025-04071-7, PMID: 40423850 PMC12116413

[29] Iyengar NM Gucalp A Dannenberg AJ Hudis CA. Obesity and Cancer mechanisms: tumor microenvironment and inflammation. J Clin Oncol. (2016) 34:4270–6. doi: 10.1200/jco.2016.67.4283, PMID: 27903155 PMC5562428

[30] Sallati I Abend Bardagi J Mendonça JA Degasperi GR. Evaluating obesity and fat cells as possible important metabolic players in childhood leukemia. J Pediatr Endocrinol Metab. (2025) 38:788–95. doi: 10.1515/jpem-2024-0448, PMID: 40294346

[31] Hong Y Li J Xu N Shu W Chen F Mi Y. Integrative genomic and single-cell framework identifies Druggable targets for colorectal Cancer precision therapy. Front Immunol. (2025) 16:1604154. doi: 10.3389/fimmu.2025.1604154, PMID: 40496862 PMC12149120

[32] An R Xiang X. Age-period-cohort analyses of obesity prevalence in us adults. Public Health. (2016) 141:163–9. doi: 10.1016/j.puhe.2016.09.021, PMID: 27931993

[33] Palmer BF Clegg DJ. The sexual dimorphism of obesity. Mol Cell Endocrinol. (2015) 402:113–9. doi: 10.1016/j.mce.2014.11.029, PMID: 25578600 PMC4326001

[34] Chooi YC Ding C Magkos F. The epidemiology of obesity. Metab Clin Exp. (2019) 92:6–10. doi: 10.1016/j.metabol.2018.09.005, PMID: 30253139

[35] Yang TW Wang CC Hung WC Liu YH Sung WW Tsai MC. Improvement in the mortality-to-incidence ratios for gastric Cancer in developed countries with high health expenditures. Front Public Health. (2021) 9:713895. doi: 10.3389/fpubh.2021.713895, PMID: 34485236 PMC8415830

[36] Ling S Zhou L Wu Y Zhang X Han W Cui L. Global, regional, and National Burden of cancers attributable to occupational risks from 1990 to 2019. J Occup Health. (2024) 66:40. doi: 10.1093/joccuh/uiae040, PMID: 39046455 PMC11378634

[37] Karupaiah T Rahman SMM Zhang J Kumar N Jamiyan B Pokharel RK. Extent and nature of television food and nonalcoholic beverage marketing in 9 Asian countries: cross-sectional study using a harmonized approach. JMIR Pediatr Parent. (2024) 7:e63410. doi: 10.2196/63410, PMID: 39630493 PMC11656118

[38] Reeve E Lamichhane P McKenzie B Waqa G Webster J Snowdon W. The tide of dietary risks for noncommunicable diseases in Pacific Islands: An analysis of population Ncd surveys. BMC Public Health. (2022) 22:1521. doi: 10.1186/s12889-022-13808-3, PMID: 35948900 PMC9364577

[39] Spear G Lee K DePersia A Lienhoop T Saha P. Updates in breast Cancer screening and diagnosis. Curr Treat Options in Oncol. (2024) 25:1451–60. doi: 10.1007/s11864-024-01271-8, PMID: 39466539

[40] Gheller A Tuttle R Oxenberg J. Breast Cancer risk, screening, and risk reduction in young females. Am Surg. (2025) 91:1178–87. doi: 10.1177/00031348251331294, PMID: 40228550

[41] Zhang M Ward J Strawbridge RJ Anderson JJ Celis-Morales C Pell JP. Genetic predisposition to adiposity, and type 2 diabetes: the role of lifestyle and phenotypic adiposity. Eur J Endocrinol. (2025) 192:549–57. doi: 10.1093/ejendo/lvaf084, PMID: 40315335 PMC12056655

[42] Kim H Kim SE Sung MK. Sex and gender differences in obesity: biological, sociocultural, and clinical perspectives. World J Mens Health. (2025) 43:126. doi: 10.5534/wjmh.250126, PMID: 40676890 PMC12505483

