# Supplementary material for: Global, regional, and national burden of high body-mass index-related cancers and associated preventable life expectancy loss from 1990 to 2021
Source: Front Nutr. 2025 Aug 19;12:1641276. doi: 10.3389/fnut.2025.1641276 (PMC12401690; doi:10.3389/fnut.2025.1641276)
Supplement: Supplementary file 1 [file Table_1.docx]

**Supplementary Table 1.Age-specific life expectancy in different regions in females in 1990**

| Age group | Global^a^ | High SDI^a^ | High-middle SDI^a^ | Middle SDI^a^ | Low-middle SDI^a^ | Low SDI^a^ | Global^b^ | High SDI^b^ | High-middle SDI^b^ | Middle SDI^b^ | Low-middle SDI^b^ | Low SDI^b^ |
| --- | --- | --- | --- | --- | --- | --- | --- | --- | --- | --- | --- | --- |
| 0 | 65.28(64.98-65.58) | 78.72(78.52-78.92) | 72.65(72.42-72.89) | 67.57(67.31-67.84) | 58.79(58.48-59.09) | 48.89(48.56-49.21) | 65.34(65.04-65.64) | 78.87(78.67-79.07) | 72.75(72.51-72.98) | 67.6(67.34-67.87) | 58.81(58.5-59.11) | 48.9(48.57-49.22) |
| 1-4 | 67.91(67.6-68.21) | 78.4(78.21-78.59) | 73.35(73.13-73.58) | 69.35(69.09-69.62) | 62.37(62.06-62.68) | 52.79(52.44-53.13) | 67.97(67.67-68.28) | 78.55(78.36-78.75) | 73.45(73.22-73.68) | 69.39(69.12-69.65) | 62.39(62.08-62.7) | 52.8(52.45-53.14) |
| 5-9 | 69.09(68.87-69.31) | 74.86(74.68-75.03) | 71.24(71.06-71.42) | 68.5(68.3-68.7) | 64.36(64.13-64.59) | 60.14(59.9-60.39) | 69.16(68.94-69.38) | 75.01(74.83-75.19) | 71.33(71.15-71.52) | 68.54(68.33-68.74) | 64.38(64.15-64.61) | 60.16(59.92-60.4) |
| 10-14 | 64.51(64.3-64.71) | 69.94(69.76-70.11) | 66.4(66.23-66.58) | 63.81(63.62-64.01) | 59.94(59.72-60.15) | 55.87(55.65-56.1) | 64.58(64.37-64.78) | 70.09(69.91-70.27) | 66.5(66.32-66.68) | 63.85(63.65-64.04) | 59.96(59.74-60.17) | 55.89(55.66-56.12) |
| 15-19 | 59.75(59.55-59.95) | 65(64.83-65.18) | 61.52(61.34-61.69) | 59.01(58.82-59.2) | 55.25(55.04-55.46) | 51.3(51.08-51.52) | 59.82(59.62-60.02) | 65.16(64.98-65.33) | 61.61(61.44-61.79) | 59.05(58.86-59.23) | 55.27(55.06-55.48) | 51.31(51.09-51.53) |
| 20-24 | 55.07(54.88-55.26) | 60.13(59.96-60.3) | 56.68(56.51-56.84) | 54.26(54.08-54.44) | 50.74(50.54-50.94) | 46.89(46.68-47.1) | 55.14(54.95-55.33) | 60.28(60.11-60.45) | 56.77(56.61-56.94) | 54.3(54.12-54.48) | 50.76(50.56-50.96) | 46.91(46.7-47.12) |
| 25-29 | 50.45(50.27-50.64) | 55.26(55.09-55.43) | 51.85(51.69-52.02) | 49.56(49.39-49.73) | 46.34(46.15-46.52) | 42.71(42.51-42.9) | 50.53(50.34-50.71) | 55.41(55.25-55.58) | 51.95(51.79-52.11) | 49.6(49.42-49.77) | 46.36(46.17-46.54) | 42.72(42.53-42.92) |
| 30-34 | 45.84(45.67-46.02) | 50.4(50.24-50.56) | 47.03(46.88-47.19) | 44.88(44.71-45.04) | 41.93(41.76-42.11) | 38.58(38.4-38.76) | 45.92(45.74-46.09) | 50.55(50.39-50.72) | 47.13(46.97-47.29) | 44.91(44.75-45.08) | 41.95(41.78-42.13) | 38.6(38.42-38.78) |
| 35-39 | 41.24(41.08-41.41) | 45.58(45.42-45.73) | 42.26(42.1-42.41) | 40.23(40.07-40.39) | 37.52(37.36-37.69) | 34.43(34.27-34.6) | 41.32(41.15-41.48) | 45.73(45.57-45.89) | 42.35(42.2-42.51) | 40.27(40.11-40.43) | 37.54(37.38-37.71) | 34.45(34.28-34.62) |
| 40-44 | 36.68(36.52-36.84) | 40.8(40.64-40.95) | 37.55(37.4-37.7) | 35.64(35.49-35.79) | 33.13(32.98-33.29) | 30.31(30.15-30.46) | 36.76(36.6-36.91) | 40.95(40.8-41.1) | 37.65(37.5-37.79) | 35.67(35.52-35.82) | 33.15(33-33.31) | 30.32(30.17-30.48) |
| 45-49 | 32.2(32.05-32.35) | 36.08(35.93-36.23) | 32.94(32.8-33.08) | 31.14(31-31.28) | 28.82(28.68-28.97) | 26.25(26.11-26.39) | 32.28(32.13-32.43) | 36.24(36.09-36.38) | 33.04(32.9-33.18) | 31.17(31.03-31.32) | 28.84(28.7-28.99) | 26.26(26.12-26.41) |
| 50-54 | 27.85(27.71-27.99) | 31.48(31.34-31.62) | 28.44(28.31-28.57) | 26.74(26.61-26.87) | 24.64(24.5-24.77) | 22.35(22.22-22.48) | 27.92(27.78-28.06) | 31.64(31.5-31.78) | 28.53(28.4-28.66) | 26.77(26.64-26.9) | 24.66(24.52-24.79) | 22.37(22.24-22.5) |
| 55-59 | 23.7(23.58-23.83) | 27.02(26.89-27.16) | 24.12(24-24.24) | 22.6(22.47-22.72) | 20.73(20.6-20.85) | 18.73(18.62-18.85) | 23.77(23.64-23.9) | 27.16(27.03-27.3) | 24.21(24.09-24.33) | 22.62(22.5-22.74) | 20.74(20.62-20.86) | 18.75(18.63-18.86) |
| 60-64 | 19.79(19.67-19.9) | 22.75(22.63-22.87) | 20.01(19.9-20.12) | 18.69(18.58-18.8) | 17.08(16.97-17.18) | 15.41(15.3-15.51) | 19.85(19.73-19.96) | 22.87(22.75-22.99) | 20.08(19.97-20.19) | 18.72(18.61-18.82) | 17.09(16.98-17.2) | 15.42(15.31-15.52) |
| 65-69 | 16.14(16.04-16.24) | 18.71(18.6-18.82) | 16.13(16.03-16.22) | 15.07(14.98-15.17) | 13.82(13.73-13.92) | 12.46(12.37-12.54) | 16.19(16.09-16.29) | 18.81(18.7-18.92) | 16.18(16.09-16.28) | 15.09(15-15.19) | 13.84(13.74-13.93) | 12.46(12.38-12.55) |
| 70-74 | 12.81(12.72-12.9) | 14.95(14.85-15.05) | 12.58(12.5-12.66) | 11.8(11.72-11.88) | 10.93(10.85-11.01) | 9.88(9.81-9.96) | 12.85(12.76-12.94) | 15.04(14.94-15.14) | 12.62(12.53-12.7) | 11.81(11.73-11.89) | 10.94(10.86-11.02) | 9.89(9.82-9.96) |
| 75-79 | 9.95(9.87-10.02) | 11.54(11.45-11.63) | 9.49(9.42-9.56) | 9.05(8.98-9.11) | 8.58(8.52-8.65) | 7.78(7.72-7.84) | 9.98(9.9-10.05) | 11.6(11.52-11.69) | 9.52(9.45-9.59) | 9.05(8.99-9.12) | 8.59(8.52-8.66) | 7.79(7.73-7.85) |
| 80-84 | 7.52(7.45-7.58) | 8.66(8.58-8.74) | 6.97(6.91-7.03) | 6.76(6.7-6.82) | 6.67(6.61-6.73) | 6.08(6.03-6.13) | 7.54(7.47-7.61) | 8.7(8.62-8.79) | 6.99(6.93-7.05) | 6.77(6.71-6.82) | 6.67(6.61-6.73) | 6.08(6.03-6.13) |
| ≥85 | 5.62(5.54-5.69) | 6.41(6.32-6.5) | 5.07(5.01-5.13) | 4.97(4.91-5.04) | 5.17(5.11-5.24) | 4.7(4.65-4.76) | 5.64(5.56-5.71) | 6.44(6.35-6.54) | 5.08(5.02-5.15) | 4.98(4.92-5.04) | 5.17(5.11-5.24) | 4.7(4.65-4.76) |

^a^ : all cause death; ^b^ : remove high BMI death
