# Supplementary material for: Global, regional, and national burden of high body-mass index-related cancers and associated preventable life expectancy loss from 1990 to 2021
Source: Front Nutr. 2025 Aug 19;12:1641276. doi: 10.3389/fnut.2025.1641276 (PMC12401690; doi:10.3389/fnut.2025.1641276)
Supplement: Supplementary file 2 [file Table_2.docx]

**Supplementary Table 2.Age-specific life expectancy in different regions in males in 1990**

| Age group | Global^a^ | High SDI^a^ | High-middle SDI^a^ | Middle SDI^a^ | Low-middle SDI^a^ | Low SDI^a^ | Global^b^ | High SDI^b^ | High-middle SDI^b^ | Middle SDI^b^ | Low-middle SDI^b^ | Low SDI^b^ |
| --- | --- | --- | --- | --- | --- | --- | --- | --- | --- | --- | --- | --- |
| 0 | 60.63(60.34-60.91) | 71.91(71.7-72.12) | 65.93(65.69-66.17) | 63.15(62.89-63.42) | 56.94(56.66-57.23) | 46.52(46.21-46.84) | 60.66(60.37-60.94) | 71.98(71.77-72.19) | 65.97(65.73-66.21) | 63.17(62.9-63.43) | 56.95(56.66-57.24) | 46.53(46.21-46.84) |
| 1-4 | 63.53(63.24-63.82) | 71.68(71.47-71.89) | 66.79(66.55-67.03) | 65.3(65.04-65.57) | 61.01(60.72-61.3) | 50.88(50.54-51.21) | 63.56(63.27-63.85) | 71.76(71.55-71.97) | 66.83(66.59-67.07) | 65.32(65.05-65.58) | 61.01(60.72-61.31) | 50.88(50.55-51.21) |
| 5-9 | 64.01(63.79-64.23) | 68.19(67.99-68.38) | 64.58(64.38-64.78) | 64.23(64.01-64.44) | 61.8(61.57-62.03) | 57.74(57.5-57.97) | 64.04(63.83-64.26) | 68.26(68.07-68.46) | 64.62(64.42-64.82) | 64.24(64.03-64.46) | 61.81(61.58-62.03) | 57.74(57.5-57.98) |
| 10-14 | 59.48(59.27-59.69) | 63.29(63.1-63.48) | 59.82(59.63-60.02) | 59.62(59.42-59.83) | 57.39(57.17-57.6) | 53.58(53.36-53.8) | 59.51(59.3-59.72) | 63.37(63.17-63.56) | 59.86(59.67-60.06) | 59.64(59.43-59.84) | 57.39(57.18-57.61) | 53.58(53.36-53.8) |
| 15-19 | 54.75(54.55-54.95) | 58.38(58.19-58.57) | 54.99(54.8-55.18) | 54.86(54.66-55.06) | 52.72(52.52-52.93) | 49.02(48.81-49.23) | 54.78(54.58-54.98) | 58.46(58.27-58.65) | 55.03(54.84-55.22) | 54.88(54.68-55.08) | 52.73(52.53-52.94) | 49.03(48.81-49.24) |
| 20-24 | 50.16(49.97-50.36) | 53.66(53.47-53.84) | 50.28(50.09-50.46) | 50.24(50.05-50.43) | 48.17(47.98-48.37) | 44.82(44.62-45.02) | 50.2(50-50.39) | 53.73(53.55-53.92) | 50.32(50.13-50.5) | 50.26(50.06-50.45) | 48.18(47.99-48.38) | 44.82(44.63-45.02) |
| 25-29 | 45.63(45.45-45.81) | 48.99(48.82-49.17) | 45.63(45.45-45.8) | 45.69(45.5-45.87) | 43.71(43.52-43.9) | 40.59(40.41-40.78) | 45.66(45.48-45.84) | 49.07(48.89-49.24) | 45.67(45.49-45.84) | 45.7(45.52-45.88) | 43.72(43.53-43.9) | 40.6(40.41-40.79) |
| 30-34 | 41.09(40.92-41.27) | 44.32(44.15-44.49) | 40.99(40.82-41.16) | 41.15(40.98-41.32) | 39.27(39.09-39.44) | 36.28(36.11-36.46) | 41.13(40.95-41.3) | 44.4(44.23-44.56) | 41.03(40.86-41.2) | 41.17(40.99-41.34) | 39.28(39.1-39.45) | 36.29(36.12-36.46) |
| 35-39 | 36.62(36.45-36.78) | 39.68(39.52-39.84) | 36.44(36.27-36.6) | 36.68(36.52-36.85) | 34.88(34.71-35.05) | 32.06(31.9-32.22) | 36.65(36.48-36.81) | 39.76(39.6-39.92) | 36.48(36.31-36.64) | 36.7(36.53-36.86) | 34.89(34.72-35.06) | 32.06(31.9-32.23) |
| 40-44 | 32.23(32.07-32.38) | 35.1(34.95-35.25) | 31.99(31.83-32.14) | 32.3(32.14-32.45) | 30.6(30.45-30.76) | 27.96(27.81-28.11) | 32.26(32.11-32.42) | 35.18(35.02-35.33) | 32.02(31.87-32.18) | 32.31(32.16-32.46) | 30.61(30.45-30.77) | 27.97(27.81-28.12) |
| 45-49 | 27.97(27.83-28.12) | 30.6(30.45-30.74) | 27.7(27.56-27.85) | 28.04(27.9-28.18) | 26.48(26.33-26.62) | 24.05(23.91-24.19) | 28(27.86-28.15) | 30.67(30.53-30.82) | 27.74(27.6-27.88) | 28.05(27.91-28.19) | 26.48(26.34-26.63) | 24.05(23.91-24.19) |
| 50-54 | 23.89(23.76-24.02) | 26.26(26.13-26.39) | 23.59(23.46-23.72) | 23.92(23.79-24.05) | 22.53(22.4-22.67) | 20.35(20.23-20.48) | 23.92(23.79-24.05) | 26.33(26.2-26.47) | 23.63(23.5-23.76) | 23.93(23.8-24.06) | 22.54(22.41-22.67) | 20.36(20.23-20.48) |
| 55-59 | 20.1(19.98-20.22) | 22.15(22.03-22.27) | 19.82(19.7-19.93) | 20.08(19.96-20.2) | 18.91(18.79-19.03) | 17(16.89-17.12) | 20.13(20.01-20.25) | 22.22(22.09-22.34) | 19.85(19.73-19.97) | 20.09(19.97-20.21) | 18.92(18.8-19.04) | 17.01(16.9-17.12) |
| 60-64 | 16.6(16.49-16.7) | 18.35(18.24-18.46) | 16.3(16.2-16.4) | 16.51(16.4-16.61) | 15.61(15.51-15.72) | 13.97(13.87-14.07) | 16.62(16.52-16.73) | 18.41(18.3-18.52) | 16.33(16.23-16.43) | 16.52(16.41-16.62) | 15.62(15.51-15.73) | 13.97(13.87-14.07) |
| 65-69 | 13.44(13.35-13.53) | 14.87(14.77-14.96) | 13.12(13.03-13.21) | 13.26(13.17-13.34) | 12.76(12.67-12.85) | 11.31(11.23-11.4) | 13.46(13.37-13.55) | 14.92(14.82-15.01) | 13.14(13.06-13.23) | 13.26(13.17-13.35) | 12.76(12.67-12.86) | 11.32(11.23-11.4) |
| 70-74 | 10.63(10.55-10.7) | 11.75(11.67-11.83) | 10.22(10.15-10.29) | 10.36(10.29-10.44) | 10.31(10.23-10.38) | 9.1(9.03-9.17) | 10.64(10.57-10.72) | 11.79(11.71-11.87) | 10.24(10.17-10.31) | 10.37(10.29-10.44) | 10.31(10.23-10.39) | 9.1(9.03-9.17) |
| 75-79 | 8.24(8.18-8.31) | 9.01(8.94-9.08) | 7.74(7.69-7.8) | 7.99(7.93-8.05) | 8.23(8.16-8.3) | 7.28(7.22-7.34) | 8.26(8.19-8.32) | 9.04(8.97-9.11) | 7.76(7.7-7.81) | 8(7.94-8.06) | 8.23(8.17-8.3) | 7.28(7.22-7.34) |
| 80-84 | 6.27(6.22-6.32) | 6.79(6.73-6.84) | 5.72(5.68-5.77) | 6.03(5.98-6.08) | 6.54(6.49-6.6) | 5.8(5.75-5.85) | 6.28(6.23-6.33) | 6.81(6.75-6.87) | 5.73(5.69-5.78) | 6.03(5.98-6.08) | 6.55(6.49-6.6) | 5.8(5.75-5.85) |
| ≥85 | 4.7(4.64-4.75) | 5.09(5.02-5.15) | 4.07(4.02-4.11) | 4.42(4.37-4.47) | 5.25(5.18-5.32) | 4.62(4.56-4.67) | 4.71(4.65-4.76) | 5.1(5.04-5.17) | 4.07(4.03-4.12) | 4.42(4.37-4.47) | 5.25(5.19-5.32) | 4.62(4.56-4.67) |

^a^ : all cause death; ^b^ : remove high BMI death
