# Supplementary material for: Global, regional, and national burden of high body-mass index-related cancers and associated preventable life expectancy loss from 1990 to 2021
Source: Front Nutr. 2025 Aug 19;12:1641276. doi: 10.3389/fnut.2025.1641276 (PMC12401690; doi:10.3389/fnut.2025.1641276)
Supplement: Supplementary file 3 [file Table_3.docx]

**Supplementary Table 3.Age-specific life expectancy in different regions in females in 2021**

| Age group | Global^a^ | High SDI^a^ | High-middle SDI^a^ | Middle SDI^a^ | Low-middle SDI^a^ | Low SDI^a^ | Global^b^ | High SDI^b^ | High-middle SDI^b^ | Middle SDI^b^ | Low-middle SDI^b^ | Low SDI^b^ |
| --- | --- | --- | --- | --- | --- | --- | --- | --- | --- | --- | --- | --- |
| 0 | 73.89(73.64-74.13) | 83.07(82.87-83.26) | 79.89(79.71-80.07) | 76.14(75.93-76.35) | 69.29(69.06-69.52) | 63.17(62.9-63.44) | 73.98(73.74-74.23) | 83.26(83.06-83.45) | 80.03(79.85-80.21) | 76.21(76.01-76.42) | 69.33(69.1-69.56) | 63.2(62.93-63.47) |
| 1-4 | 74.76(74.52-75) | 82.33(82.14-82.52) | 79.26(79.08-79.44) | 76.16(75.95-76.36) | 70.38(70.16-70.61) | 64.98(64.71-65.25) | 74.86(74.62-75.1) | 82.52(82.33-82.71) | 79.4(79.22-79.57) | 76.23(76.02-76.43) | 70.43(70.2-70.65) | 65.01(64.74-65.27) |
| 5-9 | 72.36(72.16-72.57) | 78.49(78.3-78.67) | 75.55(75.38-75.72) | 72.75(72.56-72.93) | 67.68(67.48-67.87) | 64.39(64.18-64.6) | 72.46(72.26-72.66) | 78.68(78.49-78.86) | 75.68(75.51-75.85) | 72.82(72.63-73.01) | 67.72(67.52-67.92) | 64.42(64.21-64.63) |
| 10-14 | 67.52(67.32-67.72) | 73.51(73.33-73.7) | 70.59(70.43-70.76) | 67.85(67.66-68.03) | 62.84(62.65-63.03) | 59.67(59.47-59.88) | 67.62(67.42-67.82) | 73.71(73.52-73.89) | 70.73(70.56-70.9) | 67.92(67.73-68.1) | 62.88(62.69-63.08) | 59.7(59.5-59.9) |
| 15-19 | 62.66(62.46-62.85) | 68.55(68.37-68.73) | 65.64(65.48-65.81) | 62.93(62.75-63.12) | 57.99(57.8-58.18) | 54.89(54.7-55.09) | 62.75(62.56-62.95) | 68.74(68.56-68.93) | 65.78(65.61-65.94) | 63.01(62.82-63.19) | 58.04(57.85-58.23) | 54.92(54.72-55.12) |
| 20-24 | 57.88(57.69-58.07) | 63.63(63.45-63.81) | 60.72(60.56-60.89) | 58.08(57.9-58.26) | 53.24(53.06-53.43) | 50.25(50.06-50.44) | 57.98(57.79-58.16) | 63.82(63.64-64) | 60.86(60.7-61.02) | 58.16(57.98-58.33) | 53.29(53.1-53.47) | 50.28(50.09-50.47) |
| 25-29 | 53.15(52.96-53.33) | 58.74(58.56-58.92) | 55.82(55.66-55.98) | 53.27(53.1-53.44) | 48.54(48.37-48.72) | 45.7(45.52-45.88) | 53.24(53.06-53.43) | 58.93(58.75-59.11) | 55.95(55.79-56.11) | 53.34(53.17-53.52) | 48.59(48.41-48.76) | 45.73(45.55-45.91) |
| 30-34 | 48.43(48.25-48.6) | 53.87(53.7-54.05) | 50.92(50.76-51.08) | 48.49(48.32-48.66) | 43.87(43.7-44.04) | 41.17(41-41.34) | 48.52(48.35-48.7) | 54.07(53.89-54.24) | 51.06(50.9-51.21) | 48.57(48.4-48.73) | 43.91(43.74-44.08) | 41.19(41.02-41.36) |
| 35-39 | 43.74(43.57-43.9) | 49.04(48.87-49.21) | 46.06(45.91-46.21) | 43.74(43.58-43.9) | 39.25(39.09-39.41) | 36.69(36.53-36.85) | 43.83(43.66-44) | 49.23(49.06-49.4) | 46.2(46.05-46.35) | 43.81(43.65-43.98) | 39.29(39.13-39.45) | 36.72(36.56-36.88) |
| 40-44 | 39.1(38.94-39.26) | 44.24(44.08-44.41) | 41.26(41.12-41.41) | 39.05(38.89-39.21) | 34.7(34.54-34.85) | 32.29(32.14-32.44) | 39.2(39.04-39.36) | 44.43(44.27-44.6) | 41.4(41.25-41.55) | 39.12(38.97-39.28) | 34.74(34.59-34.89) | 32.31(32.16-32.46) |
| 45-49 | 34.57(34.41-34.72) | 39.5(39.35-39.66) | 36.54(36.4-36.68) | 34.46(34.31-34.61) | 30.27(30.12-30.41) | 28(27.87-28.14) | 34.66(34.51-34.82) | 39.7(39.54-39.86) | 36.68(36.53-36.82) | 34.53(34.38-34.68) | 30.31(30.17-30.46) | 28.03(27.89-28.17) |
| 50-54 | 30.12(29.98-30.26) | 34.85(34.69-35) | 31.88(31.75-32.02) | 29.96(29.83-30.1) | 25.96(25.83-26.1) | 23.87(23.74-23.99) | 30.22(30.07-30.36) | 35.04(34.88-35.19) | 32.01(31.88-32.15) | 30.03(29.9-30.17) | 26.01(25.87-26.14) | 23.9(23.77-24.02) |
| 55-59 | 25.81(25.67-25.94) | 30.3(30.16-30.45) | 27.32(27.19-27.44) | 25.59(25.46-25.72) | 21.87(21.75-21.99) | 19.93(19.82-20.05) | 25.9(25.76-26.03) | 30.48(30.33-30.63) | 27.44(27.31-27.56) | 25.65(25.52-25.78) | 21.91(21.78-22.03) | 19.96(19.84-20.07) |
| 60-64 | 21.72(21.59-21.84) | 25.9(25.77-26.04) | 22.92(22.8-23.04) | 21.43(21.31-21.55) | 18.09(17.99-18.2) | 16.32(16.22-16.42) | 21.79(21.67-21.92) | 26.07(25.93-26.2) | 23.03(22.91-23.15) | 21.48(21.37-21.6) | 18.13(18.02-18.23) | 16.34(16.24-16.44) |
| 65-69 | 17.9(17.79-18.01) | 21.69(21.56-21.82) | 18.81(18.7-18.92) | 17.58(17.48-17.69) | 14.6(14.5-14.69) | 13.05(12.96-13.13) | 17.97(17.86-18.08) | 21.83(21.7-21.96) | 18.9(18.79-19.01) | 17.62(17.52-17.73) | 14.62(14.53-14.72) | 13.06(12.97-13.15) |
| 70-74 | 14.38(14.28-14.48) | 17.67(17.55-17.79) | 14.96(14.86-15.06) | 14.01(13.91-14.1) | 11.54(11.45-11.62) | 10.18(10.11-10.25) | 14.43(14.34-14.53) | 17.79(17.67-17.91) | 15.03(14.93-15.12) | 14.04(13.94-14.13) | 11.55(11.47-11.63) | 10.19(10.12-10.26) |
| 75-79 | 11.23(11.14-11.32) | 13.9(13.78-14.01) | 11.49(11.41-11.58) | 10.83(10.75-10.91) | 8.96(8.89-9.03) | 7.81(7.75-7.87) | 11.27(11.18-11.36) | 13.99(13.88-14.11) | 11.54(11.46-11.63) | 10.85(10.77-10.93) | 8.97(8.91-9.04) | 7.81(7.76-7.87) |
| 80-84 | 8.46(8.38-8.55) | 10.53(10.41-10.64) | 8.4(8.32-8.48) | 8.07(8-8.14) | 6.68(6.62-6.74) | 5.73(5.68-5.77) | 8.5(8.42-8.58) | 10.6(10.49-10.71) | 8.43(8.35-8.51) | 8.08(8.01-8.16) | 6.69(6.63-6.75) | 5.73(5.69-5.78) |
| ≥85 | 6.33(6.24-6.42) | 7.66(7.53-7.78) | 6.21(6.12-6.3) | 5.99(5.91-6.07) | 5.05(4.99-5.11) | 4.1(4.06-4.15) | 6.36(6.27-6.45) | 7.72(7.59-7.84) | 6.24(6.15-6.33) | 6(5.92-6.08) | 5.06(4.99-5.12) | 4.1(4.06-4.15) |

^a^ : all cause death; ^b^ : remove high BMI death
