# Supplementary material for: Global, regional, and national burden of high body-mass index-related cancers and associated preventable life expectancy loss from 1990 to 2021
Source: Front Nutr. 2025 Aug 19;12:1641276. doi: 10.3389/fnut.2025.1641276 (PMC12401690; doi:10.3389/fnut.2025.1641276)
Supplement: Supplementary file 4 [file Table_4.docx]

**Supplementary Table 4.Age-specific life expectancy in different regions in males in 2021**

| Age group | Global^a^ | High SDI^a^ | High-middle SDI^a^ | Middle SDI^a^ | Low-middle SDI^a^ | Low SDI^a^ | Global^b^ | High SDI^b^ | High-middle SDI^b^ | Middle SDI^b^ | Low-middle SDI^b^ | Low SDI^b^ |
| --- | --- | --- | --- | --- | --- | --- | --- | --- | --- | --- | --- | --- |
| 0 | 68.08(67.84-68.32) | 77.43(77.23-77.64) | 73.05(72.85-73.24) | 69.95(69.73-70.16) | 64.41(64.18-64.64) | 58.55(58.29-58.81) | 68.15(67.91-68.4) | 77.55(77.35-77.76) | 73.12(72.92-73.32) | 69.98(69.76-70.2) | 64.43(64.2-64.65) | 58.56(58.3-58.82) |
| 1-4 | 69.09(68.85-69.33) | 76.74(76.54-76.95) | 72.49(72.3-72.69) | 70.06(69.84-70.27) | 65.63(65.41-65.85) | 60.65(60.39-60.91) | 69.17(68.93-69.41) | 76.86(76.66-77.07) | 72.56(72.37-72.76) | 70.09(69.88-70.31) | 65.65(65.42-65.87) | 60.66(60.39-60.92) |
| 5-9 | 66.63(66.42-66.83) | 72.92(72.72-73.11) | 68.81(68.62-69) | 66.67(66.47-66.87) | 62.82(62.62-63.02) | 60.02(59.82-60.23) | 66.7(66.49-66.91) | 73.04(72.84-73.24) | 68.88(68.69-69.07) | 66.71(66.51-66.91) | 62.83(62.63-63.03) | 60.03(59.82-60.24) |
| 10-14 | 61.81(61.61-62.02) | 67.95(67.76-68.15) | 63.88(63.69-64.06) | 61.8(61.6-61.99) | 58(57.81-58.2) | 55.36(55.16-55.56) | 61.89(61.68-62.09) | 68.07(67.88-68.27) | 63.95(63.76-64.13) | 61.83(61.63-62.03) | 58.02(57.83-58.22) | 55.37(55.17-55.57) |
| 15-19 | 56.96(56.76-57.16) | 63(62.8-63.19) | 58.95(58.76-59.13) | 56.91(56.71-57.1) | 53.16(52.97-53.35) | 50.59(50.4-50.79) | 57.04(56.84-57.24) | 63.12(62.92-63.31) | 59.02(58.83-59.2) | 56.94(56.75-57.14) | 53.18(52.99-53.37) | 50.6(50.4-50.8) |
| 20-24 | 52.25(52.05-52.44) | 58.15(57.96-58.35) | 54.1(53.92-54.28) | 52.14(51.95-52.33) | 48.44(48.26-48.63) | 46.05(45.86-46.23) | 52.33(52.13-52.52) | 58.28(58.09-58.47) | 54.17(53.99-54.35) | 52.18(51.99-52.37) | 48.46(48.28-48.65) | 46.06(45.87-46.24) |
| 25-29 | 47.62(47.44-47.81) | 53.42(53.24-53.6) | 49.32(49.14-49.49) | 47.49(47.31-47.67) | 43.82(43.64-44) | 41.55(41.38-41.73) | 47.7(47.51-47.89) | 53.54(53.36-53.73) | 49.39(49.21-49.56) | 47.52(47.34-47.7) | 43.84(43.66-44.02) | 41.56(41.39-41.74) |
| 30-34 | 43.03(42.85-43.21) | 48.71(48.54-48.89) | 44.57(44.4-44.74) | 42.89(42.71-43.06) | 39.25(39.08-39.42) | 37.08(36.91-37.24) | 43.11(42.93-43.28) | 48.83(48.66-49.01) | 44.64(44.47-44.81) | 42.92(42.75-43.1) | 39.26(39.09-39.43) | 37.09(36.92-37.25) |
| 35-39 | 38.51(38.34-38.68) | 44.04(43.87-44.21) | 39.91(39.75-40.08) | 38.35(38.19-38.52) | 34.78(34.62-34.94) | 32.71(32.55-32.87) | 38.59(38.42-38.75) | 44.16(43.99-44.33) | 39.98(39.82-40.14) | 38.39(38.22-38.55) | 34.8(34.64-34.96) | 32.72(32.56-32.88) |
| 40-44 | 34.08(33.92-34.24) | 39.4(39.24-39.56) | 35.36(35.21-35.52) | 33.91(33.76-34.06) | 30.41(30.26-30.56) | 28.47(28.33-28.62) | 34.16(34-34.32) | 39.52(39.36-39.68) | 35.43(35.28-35.58) | 33.94(33.79-34.1) | 30.43(30.28-30.58) | 28.48(28.33-28.63) |
| 45-49 | 29.82(29.67-29.97) | 34.84(34.68-34.99) | 30.96(30.81-31.1) | 29.61(29.47-29.75) | 26.27(26.13-26.41) | 24.47(24.33-24.6) | 29.9(29.75-30.04) | 34.95(34.8-35.11) | 31.02(30.88-31.16) | 29.64(29.5-29.79) | 26.29(26.15-26.43) | 24.48(24.34-24.61) |
| 50-54 | 25.67(25.54-25.81) | 30.38(30.24-30.53) | 26.64(26.5-26.77) | 25.42(25.29-25.55) | 22.28(22.15-22.41) | 20.64(20.52-20.77) | 25.75(25.61-25.88) | 30.5(30.35-30.64) | 26.7(26.56-26.83) | 25.45(25.32-25.58) | 22.3(22.17-22.42) | 20.65(20.53-20.77) |
| 55-59 | 21.72(21.59-21.84) | 26.09(25.96-26.23) | 22.48(22.36-22.6) | 21.42(21.3-21.54) | 18.55(18.43-18.66) | 17.08(16.98-17.19) | 21.79(21.66-21.91) | 26.2(26.07-26.34) | 22.54(22.42-22.66) | 21.44(21.33-21.56) | 18.56(18.45-18.68) | 17.09(16.98-17.2) |
| 60-64 | 18.02(17.91-18.14) | 22.04(21.92-22.17) | 18.59(18.48-18.69) | 17.65(17.55-17.76) | 15.18(15.08-15.28) | 13.86(13.77-13.95) | 18.09(17.98-18.2) | 22.14(22.02-22.27) | 18.64(18.53-18.74) | 17.67(17.57-17.78) | 15.19(15.09-15.29) | 13.86(13.77-13.96) |
| 65-69 | 14.7(14.6-14.8) | 18.26(18.15-18.37) | 15.09(15-15.19) | 14.27(14.18-14.36) | 12.16(12.07-12.24) | 11.04(10.97-11.12) | 14.75(14.66-14.85) | 18.35(18.24-18.46) | 15.13(15.04-15.23) | 14.29(14.2-14.38) | 12.17(12.08-12.25) | 11.05(10.97-11.13) |
| 70-74 | 11.68(11.6-11.77) | 14.74(14.65-14.84) | 11.88(11.8-11.96) | 11.16(11.08-11.23) | 9.58(9.51-9.65) | 8.67(8.61-8.73) | 11.73(11.65-11.81) | 14.82(14.72-14.92) | 11.91(11.83-11.99) | 11.17(11.1-11.25) | 9.59(9.52-9.66) | 8.67(8.61-8.74) |
| 75-79 | 9.02(8.96-9.09) | 11.5(11.41-11.59) | 9.02(8.96-9.08) | 8.45(8.39-8.51) | 7.45(7.4-7.51) | 6.67(6.62-6.72) | 9.06(8.99-9.12) | 11.56(11.47-11.65) | 9.05(8.98-9.11) | 8.46(8.4-8.52) | 7.46(7.4-7.51) | 6.67(6.62-6.72) |
| 80-84 | 6.69(6.63-6.74) | 8.69(8.6-8.77) | 6.45(6.39-6.5) | 6.07(6.03-6.12) | 5.58(5.53-5.62) | 4.84(4.8-4.87) | 6.71(6.66-6.77) | 8.73(8.65-8.81) | 6.46(6.41-6.51) | 6.08(6.03-6.12) | 5.58(5.53-5.62) | 4.84(4.8-4.87) |
| ≥85 | 4.77(4.71-4.83) | 6.34(6.25-6.43) | 4.44(4.39-4.49) | 4.05(4-4.09) | 4.1(4.06-4.15) | 3.33(3.3-3.36) | 4.79(4.73-4.85) | 6.38(6.29-6.47) | 4.45(4.4-4.51) | 4.05(4.01-4.1) | 4.11(4.06-4.15) | 3.33(3.3-3.36) |

^a^ : all cause death; ^b^ : remove high BMI death
